# Nitrous Oxide in Asphyxia

**Published:** 1863-04

**Authors:** Geo. J. Ziegler


NITROUS OXIDE IN ASPHYXIA.—*By Geo. J. Ziegler, M. D.*

January 4th, 1863. On my visit to the hospital I found a parturient woman, somewhat exhausted from prolonged and inadequate efforts to give birth to a child. The pains were frequent but feeble, and of a diffused, continuous, inefficient, and exhaustive character. After a change in the position of the fœtal head, which was locked on the maternal pubis, and a moderate use of brandy, morphia, and ergot, without success in procuring a natural termination of the labor, I removed the child very readily with the short forceps. It manifested no signs of life, and was apparently quite dead, the face and head being gratly cyanosed and congested with dark venous blood; the cutaneous surface cool, clamy and livid; the body and limbs perfectly flaccid; while respiration and innervation were entirely absent, there being no effort to either breathe or make the slightest movement of any kind. The pulse was not noticed in the emergency, though it is doubtful whether there was much if any cardiac action or general circulation, notwithstanding the cord pulsated for a few minutes, with too little energy, however, if blood was transmitted, to be of much benefit, as it ceased entirely sometime before any signs of returning animation were manifest, and was severed soon after, in order to facilitate manipulation. The asphyxiation was, in fact, so profound, and the general condition of the child of such a character as to present every appearance of death. Acting however, upon the supposition that molecular life might still be sufficiently active to admit of resusciation, I immediately resorted to artificial respiration, the application of ammonia to the nostrils, affusion of cold and warm water to the cutaneous surface, mechanical stimulus to the nates and other parts of the body, etc., but with very limited effect, for after continuous effort for about fifteen minutes, I had only succeeded in exciting an occasional and very feeble gasp, but very little, if any, perceptible change in the color or circulation of the blood, congestion of the head, or general condition of the body. Learning on inquiry, that a small quantity of nitrous oxide water was at hand, I directed some to be injected into the bowels of the asphyxiated child, which was done, and with apparent advantage, for soon thereafter a marked change was manifest in the improved color and distribution of the blood, by the disappearance of the cyanosed aspect and hyperæmia of the face and head, livid hue of the skin, and the establishment of healthy hæmatosis, respiration, circulation and innervation, with active contractility of the tissues and normal tonicity of the body generally, for the child speedily became and has since remained quite lively and healthy.

In another more recent case of partial asphyxiation of a neonatus, delivered also by the forceps in the same institution, the nitrous oxide water was in like manner introduced through the bowels with apparent benefit in promoting arterialization, circulation, innervation and general cell-action, contractility, and tonicity, as the child soon after its use became quite florid and lively.

To facilitate the artificial delivery of this child, the mother was placed under the influence of chloroform, from the depressing effects of which she reacted so slowly, notwithstanding the use of ammonia, affusion, etc., that it appeared necessary to employ some more efficient stimulus. I therefore directed enemata of nitrous oxide water, but before the introduction of this liquid into the bowels, she rallied sufficiently to swallow a small quantity of it, and continued its use at short intervals until she had taken about six fluid ounces with seeming benefit, as she speedily recovered from the asphyxiated condition.

In several cases of infantile inanition, with marked cyanosed hue of mucous tissue and skin, nitrous oxide water was exhibited by the mouth with seeming advantage in arterializing the blood and prolonging the patients' lives.

With regard to the dose of the nitrous oxide, the general rule for its emplyment is to use as much as is required to produce the desired effect, though in the form of the surcharged water into the bowels, and of infants especially, it may sometimes be necessary, in order to secure the requisite quantity, to retain the injected fluid by pressure upon the anus.

These cases afford additional evidence of the value of nitrous oxide in asphyxia and toxicohæmia; and sustain the facts and theories long since presented by myself, respecting the peculiar relations of this remarkable agent to the animal economy, in supplying essential chemical elements thereto, promoting organic metamorphosis, and in exerting a dynamic influence over the general processes of life.—*The Medical and Surgical Reporter.*

